# Analyzing and Decoding Natural Reach-and-Grasp Actions Using Gel, Water and Dry EEG Systems

**DOI:** 10.3389/fnins.2020.00849

**Published:** 2020-08-12

**Authors:** Andreas Schwarz, Carlos Escolano, Luis Montesano, Gernot R. Müller-Putz

**Affiliations:** ^1^Institute of Neural Engineering, Graz University of Technology, Graz, Austria; ^2^Bitbrain, Zaragoza, Spain; ^3^Departamento de Informática e Ingeniería de Sistemas (DIIS), Instituto de Investigación en Ingeniería de Aragón (I3A), Universidad de Zaragoza, Zaragoza, Spain; ^4^BioTechMed Graz, Graz, Austria

**Keywords:** electroencephalogram, Brain-Computer Interface, reach-and-grasp, movement-related cortical potential, EEG systems, mobile EEG, dry electrodes, BCI data set

## Abstract

Reaching and grasping is an essential part of everybody’s life, it allows meaningful interaction with the environment and is key to independent lifestyle. Recent electroencephalogram (EEG)-based studies have already shown that neural correlates of natural reach-and-grasp actions can be identified in the EEG. However, it is still in question whether these results obtained in a laboratory environment can make the transition to mobile applicable EEG systems for home use. In the current study, we investigated whether EEG-based correlates of natural reach-and-grasp actions can be successfully identified and decoded using mobile EEG systems, namely the water-based EEG-Versatile^*TM*^ system and the dry-electrodes EEG-Hero^*TM*^ headset. In addition, we also analyzed gel-based recordings obtained in a laboratory environment (g.USBamp/g.Ladybird, gold standard), which followed the same experimental parameters. For each recording system, 15 study participants performed 80 self-initiated reach-and-grasp actions toward a glass (palmar grasp) and a spoon (lateral grasp). Our results confirmed that EEG-based correlates of reach-and-grasp actions can be successfully identified using these mobile systems. In a single-trial multiclass-based decoding approach, which incorporated both movement conditions and rest, we could show that the low frequency time domain (LFTD) correlates were also decodable. Grand average peak accuracy calculated on unseen test data yielded for the water-based electrode system 62.3% (9.2% STD), whereas for the dry-electrodes headset reached 56.4% (8% STD). For the gel-based electrode system 61.3% (8.6% STD) could be achieved. To foster and promote further investigations in the field of EEG-based movement decoding, as well as to allow the interested community to make their own conclusions, we provide all datasets publicly available in the BNCI Horizon 2020 database (http://bnci-horizon-2020.eu/database/data-sets).

## Introduction

The ability to reach-and-grasp is imperative for mastering any actions of daily life and represents the basis of personal independence. It changes for the worse when this ability is taken away, e.g., by a motor vehicle incident, causing a traumatic spinal cord injury (SCI) at cervical level. Needless to say, affected persons, e.g., with a high SCI, seek intervention to regain basic grasping functions (Anderson, 2004; Snoek et al., 2004).

A possible way to regain natural control could be a brain-computer interface (BCI) (Wolpaw et al., 2002; Millán et al., 2010). It enables its users to potentially control any assistive device via voluntary modulation of the users’ own brain signals. Brain signals are directly recorded e.g., non-invasively via electroencephalography (EEG) at the scalp of the user and circumvent any damaged parts of the spinal cord. It has been shown that BCIs can be successfully applied for communication (Birbaumer et al., 1999; Kaufmann et al., 2014; Halder et al., 2015; Pinegger et al., 2015; Scherer et al., 2015), however, they can also be used to generate control signals for assistive devices such robotic arms (Meng et al., 2016) or even upper limb motor neuroprostheses (Pfurtscheller et al., 2003; Müller-Putz et al., 2005, 2019; Rohm et al., 2013).

Though control of the designated device could often be successfully established, a major setback was that the control strategies relied on rather abstract mental imaginations and often did not have any direct connection to the intended movement. For instance, Pfurtscheller et al. (2003) relied on the repeated imagery of foot movements and right hand motor imagery to control the study participants’ neuroprosthesis attached on the left forearm. We believe that a more natural control strategy is necessary to support an intuitive control for end users (Müller-Putz et al., 2016). Ideally, a future control paradigm consists of one singular non-repetitive task which is similar to the task that has to be performed with such a neuroprosthesis or robotic arm.

Recent investigations have shown that brain patterns of singular upper limb movements can be identified and decoded from EEGs’ low frequency time domain (LFTD) signals. These so called movement-related cortical potentials (MRCPs) (Shibasaki et al., 1980) have been shown to hold discriminable information of upper limb movements (Ofner et al., 2017), different grasps (Agashe et al., 2015; Jochumsen et al., 2016), different reach-and-grasp actions (Randazzo et al., 2015; Iturrate et al., 2018; Schwarz et al., 2018, 2019) and can even be decoded online (Ofner et al., 2019; Schwarz et al., 2020).

However, it is still unclear whether the transition from a controlled laboratory environment and its high channel density recording systems to end users’ homes utilizing small, mobile EEG systems can be made successfully. The requirements of mobile EEG systems operated at end users’ homes are manifold: They need to be (i) easy to handle with the help of a non-expert caregiver and (ii) low in cost and maintenance. From a technical aspect, their (iii) performance needs to be in the same range as their laboratory counterparts, moreover, they (iv) need to operate in a non-laboratory environment. Studies have evaluated usability and performance of emerging mobile systems and compared them to laboratory systems considered “gold standard” (Guger et al., 2012; Pinegger et al., 2016; Di Flumeri et al., 2019). Recently, Jochumsen et al. (2020a, b) evaluated not only the performance of several mobile EEG systems with respect to movement intention detection from the LFTD, but also evaluated their usability with regards to patients, relatives and therapists. Nevertheless, datasets eligible for quantifying different electrode sets are rather scarce.

One of our goals of the Horizon 2020 Project MoreGrasp^1^ was to develop a grasp neuroprosthesis for people with SCI which could be operated via a BCI at their homes. As such, we took decisive efforts in designing mobile, state-of-the-art EEG recording systems to provide MoreGrasp end users with BCI technology at their homes (Müller-Putz et al., 2019). Based on these developments, we were able to introduce two market ready recording systems: the water-based electrodes EEG-Versatile^*TM*^ and the dry electrodes EEG-Hero^*TM*^.

The goals of the current study were threefold: Firstly, we wanted to determine whether EEG based correlates of reach-and grasp actions could be extracted. Secondly, we wanted to evaluate whether the LFTD correlates could be successfully decoded and, if so, the potential performance loss due to the transition from a gel-based, gold-standard system to mobile, non gel-based EEG systems. At last, we provide a substantial dataset of 45 study participants recorded with three different EEG systems to the scientific community to foster and promote the research on EEG-based movement decoding.

For this, we assessed the feasibility of the developed recording systems when recording natural reach-and-grasp actions. We performed an experiment in which 45 able bodied participants performed self-initiated reach-and-grasp actions on objects of daily life. Fifteen participants were measured using the mobile and water-based electrodes EEG-Versatile^*TM*^ system and 15 using the dry-electrodes EEG-Hero^*TM*^ headset in an office environment. In addition, we provide the recordings of additional 15 able bodied study participants who used a gel based (gold standard) system, who performed the same tasks in a laboratory environment.

## Materials and Methods

### Participants and Recordings

In total, 45 participants took part in the experiment. They were able-bodied and right handed. All gave written informed consent and received monetary compensation for their participation.

### Gel-Based Electrodes Recordings

**FIGURE 1:**
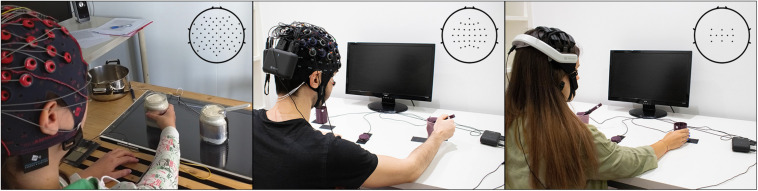
Experimental setup for the three recording systems. **Left** image shows the recordings using the gel-based system (g.tec USBamp/g.tec Ladybird system, g.tec medical engineering GmbH, Austria). **Center** image shows the water-based EEG-Versatile^*TM*^ (Bitbrain, Spain). **Right** image shows the dry-electrodes EEG-Hero^*TM*^ system (Bitbrain, Spain). Upper right corner shows the electrodes layout. EOG was recorded in the gel- and water-based electrodes systems (six additional electrodes positioned infra and superior orbital to the left and right eye and on the outer canthi).

This study was approved by the Medical University of Graz (EK: 30-439 ex 17/18). Recordings using the gel-based recording system (g.tec USBamp/g.tec Ladybird system, g.tec medical engineering GmbH, Austria) were performed at the Institute of Neural Engineering at Graz University of Technology (see Figure 1, left). We measured EEG of 15 able bodied, right handed, study participants (10 male, 5 female, aged between 15 and 30, median 26 years) with 58 active electrodes positioned over frontal, central, and parietal areas according to the 5% grid system provided by Oostenveld and Praamstra (2001). Furthermore we recorded the electrooculogram (EOG) using six additional electrodes positioned infra and superior orbital to the left and right eye and on the outer canthi. For reference we used the right earlobe and for ground the channel AFz. All signals were recorded using a sampling frequency of 256 Hz and prefiltered using an 8th order Chebyshev filter from 0.01 to 100 Hz. We used a notch filter at 50 Hz to suppress the power noise. All data was synchronized using the TOBI Signal server (Breitwieser et al., 2010). The gel based recordings provided for this study were part of another extended study incorporating also bimanual reach-and-grasp actions, which are not part of the current study. Further details can be found in Schwarz et al. (2019). Force-sensing resistor (FSR) sensors were used to record the movement onset and the grasping time point to each object. Sensor output was digitized using a battery operated Arduino microcontroller.

### Water-Based Electrodes Recordings (EEG-Versatile^*TM*^)

The recordings with the EEG-Versatile^*TM*^ system (Bitbrain, Spain) were conducted in the office environment of Bitbrain (Zaragoza, Spain), guided by personnel of the Institute of Neural Engineering, Graz University of Technology. We measured the EEG of 15 able bodied, right handed study participants (aged between 15 and 30, median 24 years; 8 females) using 32 water-based electrodes positioned over frontal, central and parietal positions (see Figure 1, center). Additionally we used six electrodes positioned infra and superior orbital and the outer canthi to measure EOG. For reference we used the left earlobe and for ground the channel AFz. These signals were recorded using a sample frequency of 256 Hz and prefiltered using a 3rd order anti-aliasing Butterworth filter with pass-band frequency from DC to 100 Hz. Photodiode sensors were used to record the movement onset and the grasping time point to each object. The three photodiodes were digitized using a Biosensing^*TM*^ amplifier (Bitbrain, Spain) at a sampling rate of 256 Hz, which was placed on the table. Time synchronization between the EEG-EOG signals and photodiodes was made via a TTL output of the Biosensing^*TM*^ amplifier. All data was streamed via Bluetooth to the computational unit using Bitbrain proprietary software, and backed-up to an internal SD card to avoid data loss due to the wireless connection.

### Dry Electrodes Recordings (EEG-Hero^*TM*^)

The recordings with the EEG-Hero^*TM*^ headset (Bitbrain, Spain) were also performed in the office environment of Bitbrain (Zaragoza, Spain), guided by personnel of the Institute of Neural Engineering, Graz University of Technology (see Figure 1, right). We measured 15 able bodied, right handed study participants (aged between 15 and 30, median 27 years; 7 females) using 11 dry electrodes located over sensorimotor areas according to the international 10/20 system (FC3, FCz, FC3, C3, C1, Cz, C2, C4, CP3, CPz, CP4). For reference and ground we used the left earlobe. These signals were recorded using a sample frequency of 256 Hz and prefiltered using a 3rd order anti-aliasing Butterworth filter with pass-band frequency from DC to 100 Hz. Photodiode sensors were used to record the movement onset and the grasping time point to each object. The three photodiodes were digitized using a Biosensing^*TM*^ amplifier (Bitbrain, Spain) at a sampling rate of 256 Hz, which was placed on the table. All data was streamed via Bluetooth to the computational unit using Bitbrain proprietary software, and backed-up to an internal SD card to avoid data loss due to the wireless connection.

### Experimental Setup and Paradigm

**FIGURE 2:**
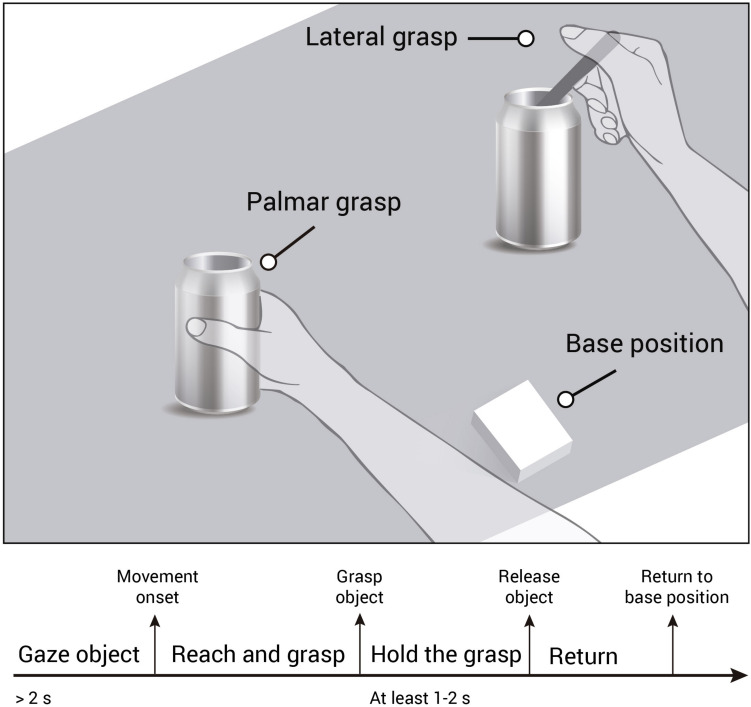
Experimental paradigm and trial timeline. Participants were instructed to gaze at the object for at least 2 s. Then they performed the reach-and-grasp action toward a jar (palmar grasp) or a jar with a spoon stuck in it (lateral grasp), and hold the grasp for at least 1–2 s. Then they returned the hand to the sensorized base position and prepared for the next trial.

All recordings were performed using the same experimental setup and followed closely the approach presented in Schwarz et al. (2019). However for gel based recordings, the experiment took place in a laboratory environment, where participants were seated in a noise and electromagnetically shielded room. For water-based and dry-electrode based recordings, the experiment took place in a non-shielded office room. Participants were seated on a chair in front of a table and instructed to rest their right hand on a sensorized base position which was positioned in front of them. On the table, we placed an empty jar and a jar with a spoon stuck in it. Both objects were in a comfortable reaching distance equidistant to the study participants’ right hand. Participants were instructed to perform reach-and-grasp actions using their right hand towards the objects placed on the table. In case of the empty jar they grasped the objects using a palmar grasp. In case of the spoon, they were instructed to grasp the spoon with a lateral grasp. Though participants performed the tasks in a self-initiated manner, we instructed them to focus their gaze on the designated object for 2 s before initiating the reach-and-grasp action. Once they completed the grasp, they held the object for at least 1–2 s (see Figure 2). When they returned their hand to the starting position, a small insert on a screen showed them the number of grasps they had already performed on the designated object. In case of the gel-based recordings, the screen was integrated in the table, for the other recording sessions, the screen was positioned in front of them. Lastly, participants paused at least for 4 s before starting a new trial (inter trial interval).

In this way we recorded 80 trials per condition (TPC) distributed over 4 runs á 20 trials. After each run, we switched the position of the objects presented on the table, so that each object was on each position equally.

We also recorded 3 min of rest at the start, after the second movement run (at half time) and at the end of the experiment, where participants were tasked to focus their gaze on a fixation point in the middle of the table. In addition, we recorded horizontal and vertical eye movements as well as blinks following the paradigms used in (Kobler et al., 2018; Schwarz et al., 2019).

### Data Preprocessing and Artifact Handling

We filtered all available data using a zero-phase 4th order Butterworth bandpass filter with a cut-off frequency of 0.3 and 60 Hz. For gel-based and water-based recordings, we used all available EEG and EOG channels and applied the extended infomax ICA algorithm on the data. We removed components associated with eye movements and blinks by visual inspection (Lee et al., 1999; Delorme and Makeig, 2004). Note that we refrained to apply an ICA algorithm on the dry-electrode based recording due to the unfavorable number of channels available (*n* = 11).

We defined a window of interest (WOI) for each movement trial of [−2 3] s with respect to the movement onset at second 0. In addition, we extracted 81 trials from the rest recordings. The rest trials had a duration of 5 s (i.e., similar to the duration of the movement trials).

Subsequently we rejected potentially artifact contaminated data by statistical parameters (Faller et al., 2012; Schwarz et al., 2015, 2018). For each participant’s data set, regardless of the recording system, we filtered the data between 0.3 and 35 Hz. We rejected trials by (1) amplitude threshold (amplitude exceeds 125 μV), (2) abnormal joint probability, and (3) abnormal kurtosis by threshold of four times the standard deviation. Trials marked for rejection were excluded from subsequent analysis. As a result, the following trials were rejected for each system: Gel-based sensors (16.0%), Water-based sensors (13.2%), and Dry sensors (8.5%).

### Power Spectral Density and Time Frequency Analysis

For each study participant, we applied a common average reference (CAR) filter on the preprocessed EEG data.

For calculating the power spectral density (PSD) estimates, we epoched all trials from (0 1.5) s with respect to the movement onset. Using Welch’s method of an overlapping segment averaging estimator, we calculated the PSD using a 1 s window and 25% overlap. We calculated the PSD average per condition and a confidence interval using non-parametric t-percentile bootstrap statistics (alpha = 0.05). To obtain the grand-average PSD, we calculated the mean over the participant-specific average and its respective confidence intervals.

For the time frequency analysis we calculated event-related (de)synchronization (ERD/S) maps in the range from 2 to 40 Hz (1 Hz resolution) as shown by Graimann et al. (2002). The analysis was performed for each movement condition separately using a specific reference interval of (−2 −1) s with respect to the movement onset. To obtain grand-average ERD/S maps (Pfurtscheller and Lopes da Silva, 1999), we calculated for each frequency bin the mean over the participants ERD/S time points and calculated confidence intervals using non-parametric t-percentile bootstrap statistics (alpha = 0.05). The resulting ERD/S maps show only the significant time-frequency points per recording system.

### Movement-Related Cortical Potentials

We resampled all preprocessed EEG signals to 16 Hz to save computational load and applied a CAR filter. Thereafter, we applied a 4th order, zero-phase Butterworth lowpass filter with a cut-off frequency of 3 Hz. To allow meaningful comparison across study participants we introduced a normalization step: For each participant, we calculated the global field power (GFP) as the standard deviation across all channels and normalized all scalp potentials by the average GFP of the rest condition (Skrandies, 1990). We epoched all movement trials and the rest recordings according to the WOI (−2 3) s and calculated condition specific averages. In addition, we determined a 95% confidence interval for each condition using non-parametric t-percentile bootstrap statistics. We accumulated a grand average per EEG system by calculating the mean over the participant-specific averages.

### Multiclass Single-Trial Classification

The classification approach follows closely the approach presented in Schwarz et al. (2019) and is adapted to the current data set. We resampled all preprocessed EEG signals to 16 Hz to save computational load and applied a CAR filter. Thereafter, we applied a 4th order, zero-phase Butterworth lowpass filter with a cut-off frequency of 3 Hz.

For each study participant, regardless of the recording system, we divided all preprocessed trials of the movement conditions as well as the rest condition in a calibration set, which consisted of the first 66% of all recorded TPC and an unseen test set consisting of the remaining 34% of all recorded TPC.

Using the calibration data set, we assessed the best time point in terms of classification accuracy for training a classification model within the WOI. For that we used a 10 times five fold cross validation approach to divide the calibration set into training and evaluation sets. For each time point within the WOI, we trained an individual shrinkage based linear discriminant classification model (sLDA) (Blankertz et al., 2011). As features, we took nine amplitude values of all available EEG channels (Gel: 58, Water: 32, Dry: 11), from the preceding second of the actual time point in causal steps of 0.125 s (−1:0.125:0) s. This yielded in total 522 features (9 × 58 channels) for the gel-based setup, 288 features (9 × 32 channels) for the water-based setup, and 99 (9 × 11 channels) for the dry electrodes setup. This classification approach was applied on each time point within the WOI yielding in 80 classification models (16 time points × 5 s WOI). To assess the best training time point, we averaged the performance results of all calculated folds and chose the time point with the best average performance. The adjusted chance level was at 45.8% [adjusted Wald interval, alpha = 0.05, (Breitwieser et al., 2012; Müller-Putz et al., 2008)] and corrected for multiple comparisons (*n* = 80 time points) using Bonferroni correction.

Thereafter, we applied the best performing classification model on previously unseen test data, using the same preprocessing pipeline as before. In this case, the adjusted chance level for the test set lies at 42.8% (adjusted Wald interval, alpha = 0.05). We show the mean classification performance of all trials over the whole WOI.

In addition, we repeated this classification approach for gel- and water-based recording systems with a subset of 11 channels covering the sensorimotor electrode positions of the dry electrodes system (EEG-Hero^*TM*^).

## Results

### Gel Based Recordings

**FIGURE 3:**
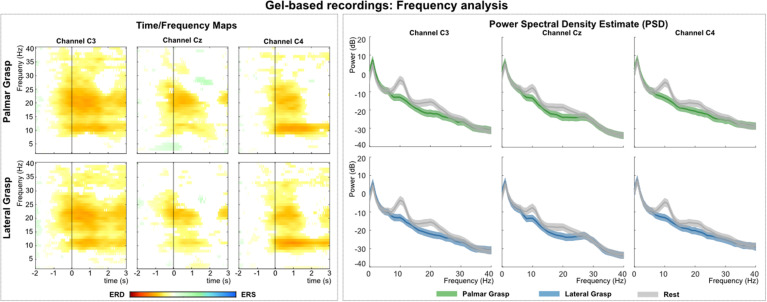
Frequency analysis of the gel-based recordings. **(Left)**: Grand average of the time-frequency maps (Graimann et al., 2002) for movement conditions for positions C3, Cz, and C4 with respect to the reference period (–2 –1) s. The black vertical line represents the movement onset. Hot colors show significant ERD (cold colors represent significant relative increase in power [event-related synchronization (ERS)]. Significant differences with respect to the reference period were calculated using non-parametric t-percentile bootstrap statistics (alpha = 0.05). **(Right)**: Grand average of the PSD calculation of the reaching phase [0 1.5] s. Colored lines represent the PSDs of the movement conditions, gray lines show the PSD of the rest condition. The shaded areas show 95% confidence intervals which were calculated using non-parametric t-percentile bootstrap statistics (alpha = 0.05).

Figure 3 (left) shows the grand average time-frequency maps of the gel-based recordings for channels C3, Cz, and C4 with respect to a reference interval (−2 −1) s prior to the movement onset. Significant ERD can be seen on the three channels, most prominent in alpha (8–12 Hz) and beta frequency range (∼20 Hz). Alpha (mu) activity shows the most prominent ERD on the ipsilateral side to the executing hand, whereas beta activity shows the most prominent ERD on the contralateral side. Figure 3 (right) shows the PSD estimates of the reaching phase (0 1.5) s for channels C3, Cz, and C4 for both the movement conditions and the rest condition. When comparing the movement conditions to the rest condition, significant power decreases can be observed mainly in the alpha and beta range. This power decrease is stronger on the contralateral side to the executing hand, especially for the beta frequency range.

**FIGURE 4:**
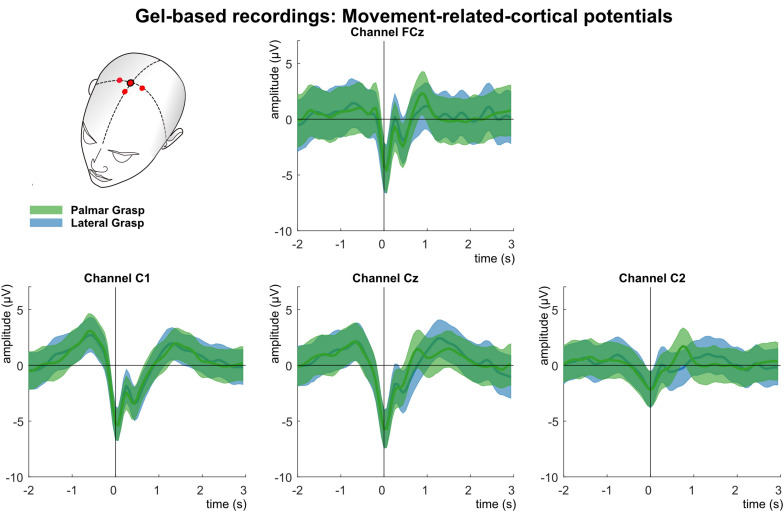
Movement related cortical potentials (MRCPs) of the gel-based recordings. Grand averages (bold lines) and 95% confidence intervals (shaded areas) for palmar (green) and lateral (blue) grasp conditions. Channels shown are FCz, C1, Cz, and C2. The black perpendicular line represents the movement onset.

Analysis of the MRCPs in the LFTD (see Figure 4) shows the grand average for the palmar and lateral grasp conditions. A negative deflection (Bereitschaftspotential, Shibasaki et al., 1980) can be observed (time = 0 s), which starts up to one second before the movement onset. This deflection is pronounced strongest first over the central motor cortex at channel position Cz, and contralateral to the executing right hand second. About 300 ms after the movement onset, a positive deflection (reafferent potential) can be observed. Thereafter, around 1 s after the movement onset, a second positive peak occurs before the potential returns to baseline. On group level, no significant differences between movement conditions could be observed.

**FIGURE 5:**
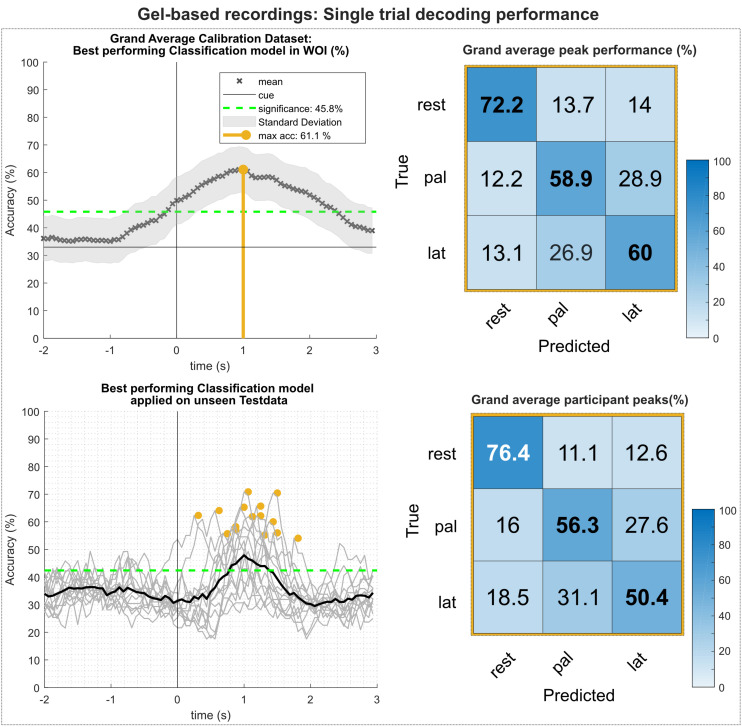
Single trial decoding performance of the gel-based recordings. **(Top left)** Grand average for the calibration data set. Black crosses show the mean performances for the designated time point. On average, best classification performance could be reached 1 s after the movement onset (perpendicular golden line). **(Top right)** Row wise normalized confusion matrix for grand average peak performance. **(Bottom left)** Participant-specific classification results (gray lines) and grand average (black bold line) of the best performing classification model applied on the unseen test data set. Golden dots show participant-specific peak performances. **(Bottom right)** Row wise normalized confusion matrix for the individual peak performances.

Figure 5 summarizes the results of the single trial multiclass decoding of both movement conditions and the rest condition (gel-based recordings). Figure 5 (top) shows the grand average obtained on the calibration data set and the corresponding confusion matrix of the grand average peak performance. On average, highest calibration performance could be reached about 1 s after the movement onset with an average peak accuracy of 61.1%. This is lower than the average of the participant-specific (63.9%) peak performance, since the time of peak performance varies between participants. Analysis of the confusion matrix shows that the true positive rate (TPR) for the rest condition is highest with 72.2%, exceeding the TPRs of the movement conditions by more than 12%. In contrast, false positive rates (FPR) for movement versus movement conditions are between 26 and 29%, exceeding FPRs of movement versus rest conditions more than twice. Figure 5 (bottom) shows the results of the participant-specific best performing classification models applied on the previously unseen test data set and its corresponding confusion matrix of the grand average of the peak performances. Participant-specific peak performances reached on average 61.3% around 1.1 s after the movement onset. The corresponding confusion matrix shows again higher TPRs for classification of the rest versus movement conditions and are within the same range as the results of the calibration data set. However, TPRs for movement versus movement conditions decreased, especially for the lateral grasp condition. Table 1 depicts the participant-specific classification results.

**TABLE 1:** Participant-specific classification results of the gel-based recordings.

	Calibration set			Test set		
#	Peak (%)	STD (%)	Time (s)	Peak (%)	STD (%)	Time (s)
G01	71.2	7.6	0.2	62.3	8.2	0.3
G02	57.0	8.6	1.1	65.7	8.9	1.2
G03	57.0	7.6	1.7	54.1	7.5	1.8
G04	73.6	8.0	0.9	57.1	8.6	0.8
G05	59.9	6.8	1.3	62.2	10.4	1.2
G06	56.4	7.6	0.7	55.7	7.1	0.7
G07	53.6	8.6	0.8	56.0	7.5	1.4
G08	71.6	6.7	1.4	70.4	10.0	1.4
G09	72.0	7.9	1.1	61.8	8.9	1.1
G10	72.0	6.5	0.9	65.3	8.6	0.9
G11	60.3	9.4	0.8	58.2	6.2	0.8
G12	64.9	7.6	1.0	70.8	10.3	1.0
G13	63.8	10.1	1.4	55.2	8.7	1.3
G14	58.1	9.7	0.4	64.1	9.7	0.6
G15	66.5	7.8	1.4	60.0	7.9	1.4
Average	63.9	8.0	1.0	61.3	8.6	1.1

### Water Based Recordings

**FIGURE 6:**
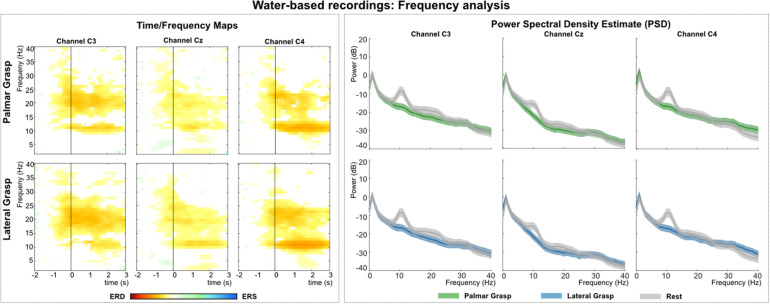
Frequency analysis of the water-based recordings. **(Left)**: Grand average of the time-frequency maps (Graimann et al., 2002) for movement conditions for positions C3, Cz, and C4 with respect to the reference period (–2 –1) s. The black vertical line represents the movement onset. Hot colors show significant ERD [cold colors represent significant relative increase in power (event-related synchronization (ERS)]. Significant differences with respect to the reference period were calculated using non-parametric t-percentile bootstrap statistics (alpha = 0.05). **(Right)**: Grand average of the PSD calculation of the reaching phase [0 1.5] s. Colored lines represent the PSDs of the movement conditions, gray lines show the PSD of the rest condition. The shaded areas show 95% confidence intervals which were calculated using non-parametric t-percentile bootstrap statistics (alpha = 0.05).

Figure 6 (left) shows the grand average time-frequency maps of the water-based recordings for channels C3, Cz, and C4 with respect to a reference interval (−2 −1) s prior to the movement onset. Significant ERD can be found for the three channels, especially in alpha (8–12 Hz) and beta (∼20 Hz) band frequencies. The differences are pronounced weakest at central electrode position Cz. Alpha (mu) activity shows the most prominent ERD on the ipsilateral side (C4), with the strongest beta at bilateral positions (C3 and C4). Figure 6 (right) shows the PSD estimates of the reaching phase (0 1.5) s for channels C3, Cz, and C4 for the movement conditions and the rest condition. Significant differences between movement conditions and rest condition can be observed on channels in the alpha band and on the contralateral side (C3 location) in the beta band.

**FIGURE 7:**
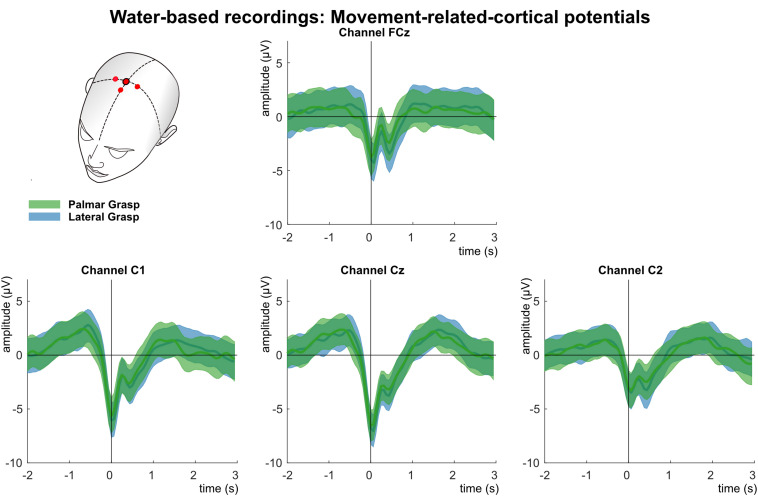
Movement related cortical potentials (MRCPs) of the water-based recordings. Grand averages (bold lines) and 95% confidence intervals (shaded areas) for palmar (green) and lateral (blue) grasp conditions. Channels shown are FCz, C1, Cz, and C2. The black perpendicular line represents the movement onset.

Figure 7 shows the analysis of the MRCPs for channels FCz, C1, Cz, and C2. Around 1 s before the movement onset, the negative deflection of the BP starts and peaks around movement onset (time = 0 s). The BP is strongest pronounced over the central electrode position first, and contralateral to the executing right hand second. It is clearly recognizable the reafferent potential around 300 ms after the movement onset followed by a second positive deflection around 1–1.5 s after the movement onset before the potentials return back to baseline. The morphologies of both movement conditions are similar and bear no significant difference on group level.

**FIGURE 8:**
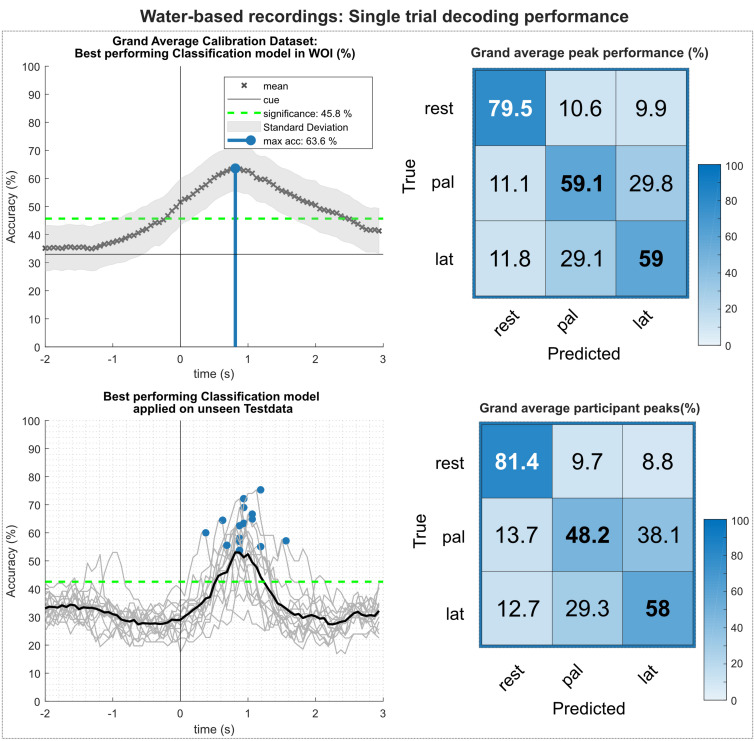
Single trial decoding performance of the water-based recordings. **(Top left)** Grand average for the calibration data set. Black crosses show the mean performances for the designated time point. On average, best classification performance could be reached 0.9 s after the movement onset (perpendicular blue line). **(Top right)** Row wise normalized confusion matrix for grand average peak performance. **(Bottom left)** Participant-specific classification results (gray lines) and grand average (black bold line) of the best performing classification model applied on the unseen test data set. Blue dots show participant-specific peak performances. **(Bottom right)** Row wise normalized confusion matrix for the individual peak performances.

Figure 8 summarizes the results of the multiclass single trial decoding. Figure 8 (top) shows the grand average obtained on the calibration data set and the corresponding confusion matrix of the grand average peak performance. Grand average peak performance reached 63.6% around 0.9 s after the movement onset. Participant-specific peak accuracies were slightly higher with 65.4%. The confusion matrix shows high TPRs for rest versus movement conditions, exceeding TPRs for movement versus movement conditions by 20%. Figure 8 (bottom) depicts the participant-specific classification results when applying the best performing classification model trained on the calibration data on the unseen test set. Participant-specific peak performance reaches on average 62.3% around 0.9 s after the movement onset. The corresponding confusion matrix shows an even more favorable TPR for rest versus movement conditions with 81.4%. However, TPRs for movement versus movement conditions decreased, especially for the palmar grasp condition. Table 2 depicts the participant-specific classification results for calibration and test set in detail.

**TABLE 2:** Participant-specific classification results of the water-based recordings.

	Calibration set			Evaluation set		
#	Peak (%)	STD (%)	Time (s)	Peak (%)	STD (%)	Time (s)
V01	68.2	7.9	0.9	69.0	11.1	0.9
V02	74.4	8.2	0.8	72.2	11.3	0.9
V03	60.1	8.9	0.8	56.9	7.7	0.8
V04	59.4	7.4	0.6	64.5	8.3	0.6
V05	60.7	7.3	0.8	58.1	8.5	0.8
V06	76.4	7.4	0.9	75.3	11.1	1.1
V07	68.3	8.2	1.3	66.7	11.1	1.0
V08	74.1	6.8	1.1	55.1	7.7	1.1
V09	59.7	7.5	0.8	63.4	11.5	0.9
V10	62.8	8.3	0.8	62.5	6.9	0.8
V11	68.0	8.0	0.9	64.9	10.3	1.0
V12	60.7	8.9	0.5	55.6	8.2	0.6
V13	60.9	7.6	0.4	60.0	6.9	0.3
V14	65.0	8.6	1.6	57.1	9.0	1.5
V15	61.9	7.7	0.9	53.7	8.5	0.8
Average	65.4	7.9	0.9	62.3	9.2	0.9

*Columns 2–4 show peak performance (%), standard deviation (%), and time of occurrence (s) with respect to the movement onset for the calibration set. Columns 5–7 show the same for the test set.*

### Dry Electrode Recordings

**FIGURE 9:**
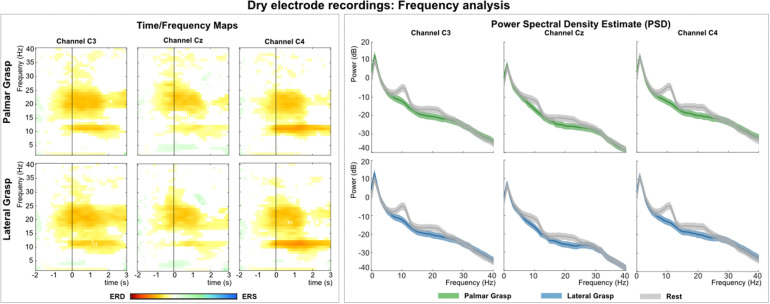
Frequency analysis of the dry-electrodes recordings. **(Left)**: Grand average of the time-frequency maps (Graimann et al., 2002) for movement conditions for positions C3, Cz, and C4 with respect to the reference period (–2 –1) s. The black vertical line represents the movement onset. Hot colors show significant ERD (cold colors represent significant relative increase in power [event-related synchronization (ERS)]. Significant differences with respect to the reference period were calculated using non-parametric t-percentile bootstrap statistics (alpha = 0.05). **(Right)**: Grand average of the PSD calculation of the reaching phase [0 1.5] s. Colored lines represent the PSDs of the movement conditions, gray lines show the PSD of the rest condition. The shaded areas show 95% confidence intervals which were calculated using non-parametric t-percentile bootstrap statistics (alpha = 0.05).

Figure 9 (left) shows the grand average time-frequency maps of the dry electrodes recordings for channels C3, Cz, and C4 with respect to a reference interval (−2 −1) s prior to the movement onset. Significant ERD can be found on the three channels, especially in alpha (8–12 Hz) and beta band (∼20 Hz). Differences are weakest on central channel location Cz. Alpha (mu) activity shows the stronger ERD on the ipsilateral side (C4), and beta presents a stronger ERD on bilateral locations (C3 and C4). Figure 9 (right) depicts the PSD estimates of the reaching phase [0 1.5] for channels FCz, C1, Cz, and C2. When looking at both movement conditions against the rest condition, a significant power decrease for the movement conditions in alpha and beta can be observed. This power decrease is more pronounced bilaterally than on the central electrode position Cz.

**FIGURE 10:**
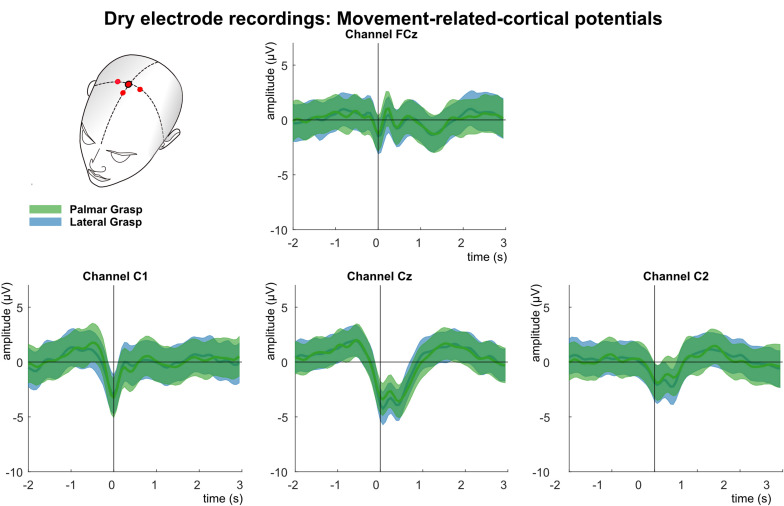
Movement related cortical potentials (MRCPs) of the dry-electrode recordings. Grand averages (bold lines) and 95% confidence intervals (shaded areas) for palmar (green) and lateral (blue) grasp conditions. Channels shown are FCz, C1, Cz, and C2. The black perpendicular line represents the movement onset.

Figure 10 shows the analysis of the MRCPs for channels FCz, C1, Cz, and C2. A negative deflection can be observed at movement onset (time = 0 s), which starts about 0.5 s before the movement onset. It is strongest on the central position first and on the contralateral side to the executing hand second. Around 300 ms after the movement onset, at least for electrode positions Cz and C2, a reafferent potential can be seen before the potential returns back to base level about 1–1.5 s after the movement onset. The morphologies of both movement conditions show no significant difference on group level.

**FIGURE 11:**
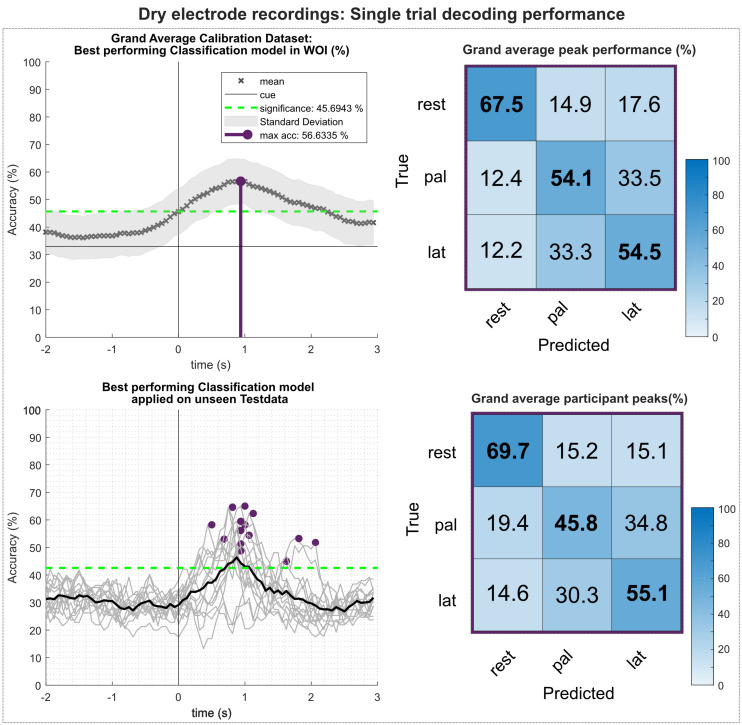
Single trial decoding performance of the dry-electrodes recordings. **(Top left)** Grand average for the calibration data set. Black crosses show the mean performances for the designated time point. On average, best classification performance could be reached 1s after the movement onset (perpendicular purple line). **(Top right)** Row wise normalized confusion matrix for grand average peak performance. **(Bottom left)** Participant-specific classification results (gray lines) and grand average (black bold line) of the best performing classification model applied on the unseen test data set. Purple dots show participant-specific peak performances. **(Bottom right)** Row wise normalized confusion matrix for the individual peak performances.

Figure 11 shows the results of the multiclass single trial decoding. Figure 11 (top) depicts the grand average performance of the calibration data set and the confusion matrix of the participant specific grand average peak performance. On average, 56.6% around 1 s after the movement onset could be reached. The participant-specific peak accuracy yielded at 58.3% and is higher due to the variation in timing of reaching peak performance of the participants. The corresponding confusion matrix shows increased TPRs for rest versus movement conditions of 67.5%, whereas TPRs for movement versus movement conditions yielded about 54%. When applying the best performing classification model on the unseen data set (Figure 11, bottom), participant-specific peak accuracies still yielded on average 56.4%. Table 3 depicts participant-specific performance results in detail.

**TABLE 3:** Participant-specific classification results of the dry-electrodes recordings.

	Calibration set			Evaluation set		
#	Peak (%)	STD (%)	Time (s)	Peak (%)	STD (%)	Time (s)
H01	63.4	8.2	1.9	53.2	7.5	1.8
H02	53.8	9.6	0.8	58.2	8.0	0.4
H03	56.4	8.2	1.1	58.2	9.2	0.9
H04	55.1	8.2	0.9	54.4	7.3	1.0
H05	54.1	8.5	0.8	48.8	6.6	0.9
H06	55.3	7.7	0.9	53.0	7.1	0.6
H07	64.5	7.5	0.6	64.6	8.1	0.8
H08	49.8	8.0	1.6	45.0	4.9	1.6
H09	65.9	8.0	0.9	62.3	7.9	1.1
H10	63.3	7.7	0.9	65.0	11.4	0.9
H11	62.3	8.4	0.7	64.6	7.8	0.8
H12	62.1	6.7	0.8	51.3	8.3	0.9
H13	56.8	6.7	1.8	51.8	8.7	2.1
H14	54.8	9.1	0.5	56.2	8.9	0.9
H15	57.5	8.2	0.7	59.5	8.5	0.9
Average	58.3	8.1	1.0	56.4	8.0	1.0

*Columns 2–4 show peak performance (%), standard deviation (%) and time of occurrence (s) with respect to the movement onset for the calibration set. Columns 5–7 show the same for the test set.*

### Comprehensive Analysis

#### Behavioral Analysis

**FIGURE 12:**
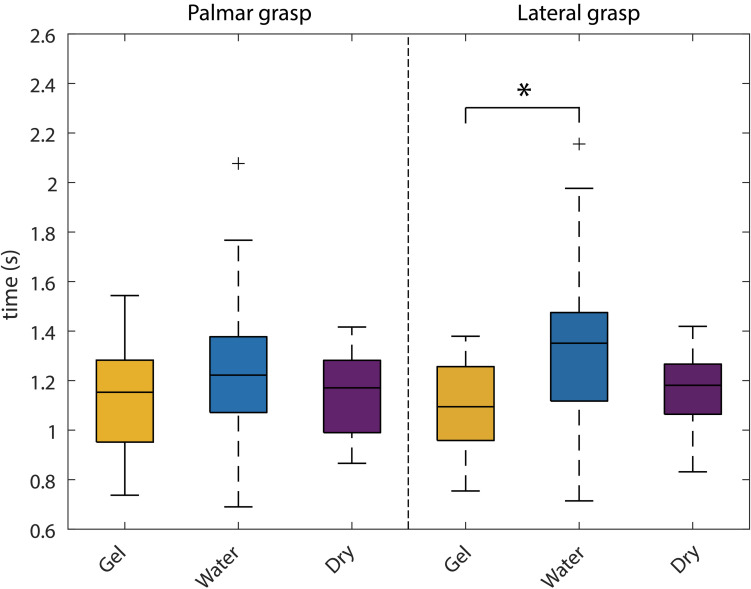
Behavioral analysis. Box-plots of the reach-and-grasp durations for all the systems. The left part shows the palmar grasp durations, whereas the right part shows the lateral grasp ones. Statistical comparisons were carried out for each grasp type separately and significant differences are marked with an asterisk.

We analyzed the duration of the reach-and-grasp actions (see Figure 12). The time information was provided by the instrumentalized objects and extracted from all trials. Then, for each participant and grasp type (palmar, lateral) the average duration was calculated. We were interested in testing, for each grasp type, the possible time differences among the three recording systems. To do so, we computed two separate one-way ANOVAs (for each grasp type) with three levels (gel, water, dry).

A significant effect was found in the lateral grasp [*F*(2,42) = 4.6; *p* = 0.016]. *Post hoc* pairwise multiple comparison tests using the Tukey–Kramer criterion showed a significant effect between the gel- and water-based recordings (*p* = 0.015).

#### Performance Analysis

We used a one-way ANOVA to compare the differences in classification accuracies among the three EEG recordings. We used separate ANOVAs for the training and test data. We found a significant effect in the training data [*F*(2,42) = 5.86; *p* = 0.006]. *Post hoc* pairwise comparisons revealed a significant performance decrease in the dry-electrodes recordings with respect to the gel- (*p* = 0.037) and water-based recordings (*p* = 0.006). Similarly, we found a significant effect in the test data [*F*(2,42) = 4.14; *p* = 0.023], and *post hoc* pairwise comparisons revealed a significant performance decrease in the dry-electrodes recordings with respect to the gel- (at statistical trend, *p* = 0.08) and water-based (*p* = 0.026) recordings.

Finally, we repeated the previous statistical analysis with the classification accuracies obtained when only using the sensorimotor channels for classification (*n* = 11). The ANOVAs testing showed no significant effects (in either training or test data) among the three EEG recordings. Participant specific performance results can be found in the Supplementary Tables S1, S2.

## Discussion

This study confirmed that EEG based correlates of reach-and-grasp actions can be successfully identified from the LFTD and time-frequency domain using the water-based EEG-Versatile^*TM*^ system and the dry-electrodes EEG-Hero^*TM*^ headset. In addition, we provided a gel-based recording in a laboratory environment (gold standard), which followed the same experimental parameters. In a single-trial multiclass based decoding approach, which incorporated both movement conditions and rest, we could show that the LFTD correlates were also decodable. Grand average peak accuracy calculated on unseen test data yielded for the water-based electrode system 62.3% (9.2% STD), whereas for the dry-electrodes headset reached 56.4% (8% STD). For the gel-based electrode system 61.3% (8.6% STD) could be achieved. The adjusted chance level for this decoding approach was 45.7%, adjusted Wald interval, alpha = 0.05 (Müller-Putz et al., 2008; Breitwieser et al., 2012).

A quantifying comparison between the individual systems is hardly possible due to inter-subject variations and technical factors such as the number of channels for calculating the spatial filters in preprocessing (Gel: 58; Water: 32; Dry: 11) that might influence the outcome. Taking this consideration into account, the gel-based and the water-based system yielded comparable decoding performances and, despite the decreased number of channels of the dry-electrodes headset, the average performance decreased only by less than 6%. Apart from these investigations, we leave it open to the reader to compare systems. For this we provide the complete data sets of all recordings so that the interested community can make their own conclusions.

### Time Frequency and PSD Analysis

Calculated ERD/S maps (Graimann et al., 2002) show significant relative power changes for all recording systems. In general, the relative power decrease (ERD) starts already up to 1 s before the actual movement onset and is most prominent in the alpha (mu) and beta frequency bands (Andrew and Pfurtscheller, 1995; Florian and Pfurtscheller, 1995). For the grand average of the gel-based recordings this power decrease is also pronounced stronger, i.e., in terms of frequency range on the contralateral side to the executing right hand than on central or ipsilateral locations. Gel-based recordings showed a more pronounced ERD on the contralateral side in beta frequency bands, which is an expected effect reported in numerous studies (Pfurtscheller and Lopes da Silva, 1999; Pfurtscheller and Neuper, 2001; Müller-Putz et al., 2010). However, this phenomenon was not visible for the water-based and dry electrodes, rather showing a pronounced bilateral ERD (Zaepffel et al., 2013). Moreover, the ERD, especially in the alpha band around 8–12 Hz is pronounced stronger on the ipsilateral side in all three types of recordings (see Supplementary Figure S1). The grand average results of the PSD analysis show for all three investigated systems significant power decreases for movement conditions when compared to the rest condition. These differences manifest again in the alpha and beta band range and are pronounced strongest on the contralateral side for the gel- and water-based recordings, thus indicating a lateralization effect measured in absolute power. Regarding the dry electrodes, this lateralization phenomenon was not found as a similar power decrease was measured in both bilateral sides.

### Movement-Related Cortical Potentials

Analysis of the MRCPs reveal on a grand average basis a strong similarity between the gel-based and the water-based recordings. Around 1 s before the movement onset a negative deflection can be seen, most pronounced over the central motor cortex (Bereitschaftspotential) (Kornhuber and Deecke, 1964; Shibasaki et al., 1980; Shibasaki and Hallett, 2006). This deflection reaches its peak at the movement onset (time = 0 s). It is strongest over central channel Cz and on the contralateral side to the executing right hand. For both systems, a reafferent positive potential around 300 ms after the movement onset can be observed. It has already been found in previous studies concerning reach-and-grasp actions (Schwarz et al., 2018, 2019). Around 1–1.5 s, a second positivity occurs before the potentials return to baseline. In contrast, the MRCPs of the dry electrode recordings are on grand average smaller and their characteristics, such as the BP or the reafferent potential, although clearly identifiable, are attenuated in comparison.

### Single-Trial Decoding

The offline classification followed closely the approach initially described in Schwarz et al. (2019) and was primarily designed to simulate a BCI scenario. Using 66% of all available data, we attempted to find the best performing classification model within a WOI. Thereafter, we applied the best performing model on the previously unseen last third of the recorded trials. Regardless of the recording system, all study participants scored significantly higher than the adjusted chance level (calibration set: 45.8%; test set: 42.8%) on both calibration and test. Comprehensive statistical analysis of the participant-specific peak performances showed no significant differences between gel-based and water-based recordings in both sets. Regarding the dry-electrodes headset, a significant decreased performance was found in comparison to the gel- and water-based systems on the calibration set, as well as to the water-based system on the test set.

This decrease in performance for the dry-electrode system was not unexpected, since the number of available electrodes in the dry electrodes (*n* = 11) is many times smaller than for gel-based (*n* = 58) or the water-based (*n* = 32) systems. In a previous study we have already investigated the effect of decreasing the number of (gel based) electrodes available for decoding (Schwarz et al., 2018): We could show that the difference in performance between 61 gel-based electrodes (covering frontal, central, and parietal areas of the scalp) and only 25 gel-based covering sensorimotor areas is minimal. However, further reducing the available electrodes to 15 led to a performance decrease comparable to the dry-electrodes recordings in the current study.

This goes in line with our second classification approach, where we used only the same 11 electrodes in all recording systems, positioned over sensorimotor areas. For both gel-based and water-based electrode systems, the performance was in the same range as for the dry-electrodes headset (see Supplementary Tables S1, S2). No significant differences in performance between recording systems could be found anymore. Unfortunately, a direct comparison to other reach-and-grasp studies such as (Agashe et al., 2015; Randazzo et al., 2015; Iturrate et al., 2018) is difficult due significant differences in experimental setup and paradigm and hence cannot be made in a serious manner.

### Corresponding Data Sets

The current manuscript is accompanied by a data set of in total 45 study participants, 15 per EEG system. In addition to reproducibility, these datasets will allow analyses beyond the basic analysis steps presented in this manuscript and we encourage the scientific community to try and evaluate new approaches. The datasets are publicly available in the BNCI Horizon 2020 database^2^.

### Study Limitations

In the current study, we investigated whether EEG-based correlates of reach-and-grasp actions can be successfully identified and decoded from three different EEG systems. However, due to differences in the amount of available EEG and EOG channels, preprocessing and artifact handling could not be performed uniformly. We applied an extended Infomax algorithm on gel-based and water-based recordings and removed ocular based components by visual inspection. This approach could not be performed on the dry electrode recordings, due to the unfavorable number of channels and subsequent number of ICA components, which did not allow a clean separation between ocular and brain activity. Due to the multicentric design of the study, we did not perform the evaluation of the EEG systems on one participant population. Instead, we performed the experiments for each EEG system on an independent group of 15 study participants. Furthermore, the object positions between the gel-based and the water-dry electrodes systems were not exactly replicated and is considered a minor deviation from the original experimental protocol.

## Conclusion

We presented an EEG dataset on natural reach-and-grasp actions recorded with three different EEG systems – gel-based, water-based and with dry electrodes.

The accompanying study confirmed that reach-and-grasp actions can be successfully identified from MRCPs and time-frequency domain using a water-based EEG-Versatile^*TM*^ system and a dry electrodes EEG-Hero^*TM*^ headset. In addition, we provided results from a gel-based recording in a laboratory environment (gold standard), which followed the same experimental parameters.

In a single-trial multiclass based decoding approach, which incorporated both movement conditions and rest, we could show that the MRCPs were also decodable. Although a quantifying comparison between the individual systems is hardly possible due to inter-subject variations and technical factors such as the different number of channels among systems, the gel-based and the water-based system yielded comparable decoding performances. Despite the decreased number of channels of the dry electrodes recordings, the average performance decreased only by less than 6%. Apart from these investigations, we also provide the complete data sets of in total 45 study participants so that the interested community can make their own conclusions. The data set is open access and available at the BNCI Horizon 2020 data base^2^.

## Data Availability Statement

The datasets presented in this study can be found in online repositories. The names of the repository/repositories and accession number(s) can be found at: http://bnci-horizon-2020.eu/database/data-sets.

## Ethics Statement

The studies involving human participants were reviewed and approved by the Medical University of Graz (EK: 30-439 ex 17/18). The patients/participants provided their written informed consent to participate in this study.

## Author Contributions

AS and CE recorded and analyzed the data. AS wrote the original draft. All authors designed the study together and were commited in editing and reviewing the manuscript.

## Conflict of Interest

CE and LM were employed by Bitbrain, Zaragoza, Spain. The remaining authors declare that the research was conducted in the absence of any commercial or financial relationships that could be construed as a potential conflict of interest.

## Footnotes

1 www.moregrasp.eu

2 http://bnci-horizon-2020.eu/database/data-sets
