# Supplementary material for: Analyzing and Decoding Natural Reach-and-Grasp Actions Using Gel, Water and Dry EEG Systems
Source: Front Neurosci. 2020 Aug 12;14:849. doi: 10.3389/fnins.2020.00849 (PMC7438923; doi:10.3389/fnins.2020.00849)
Supplement: Supplementary file 1 [file Data_Sheet_1.pdf]

Supplementary Material

1 ERDS time-frequency maps: lateralization

This paper reports grand average time-frequency maps for the three different recording systems and the two reach-and-grasp (palmar and lateral grasp) movement conditions, specifically showing the event-related (de)synchronization (ERD/S) using a specific reference interval of [-2 -1] s. In the results section we have provided a qualitative analysis of the ERDs and its lateralization, i.e., the difference in desynchronization between contralateral and ipsilateral motor areas.

We below provide a quantitative analysis of the lateralization that measures the difference in ERD between C3 and C4 sensors (Figure S1). First, we determined three frequency regions of interest: alpha (mu, 8-12 Hz), low beta (16-24 Hz) and high beta (24-30 Hz). For each participant and electrode, the mean ERD value was calculated for every region of interest in the movement interval of [0 1.5] s relative to the movement onset. Finally, for each participant, we calculated the lateralization as the difference between the ERD values in C3 and C4.

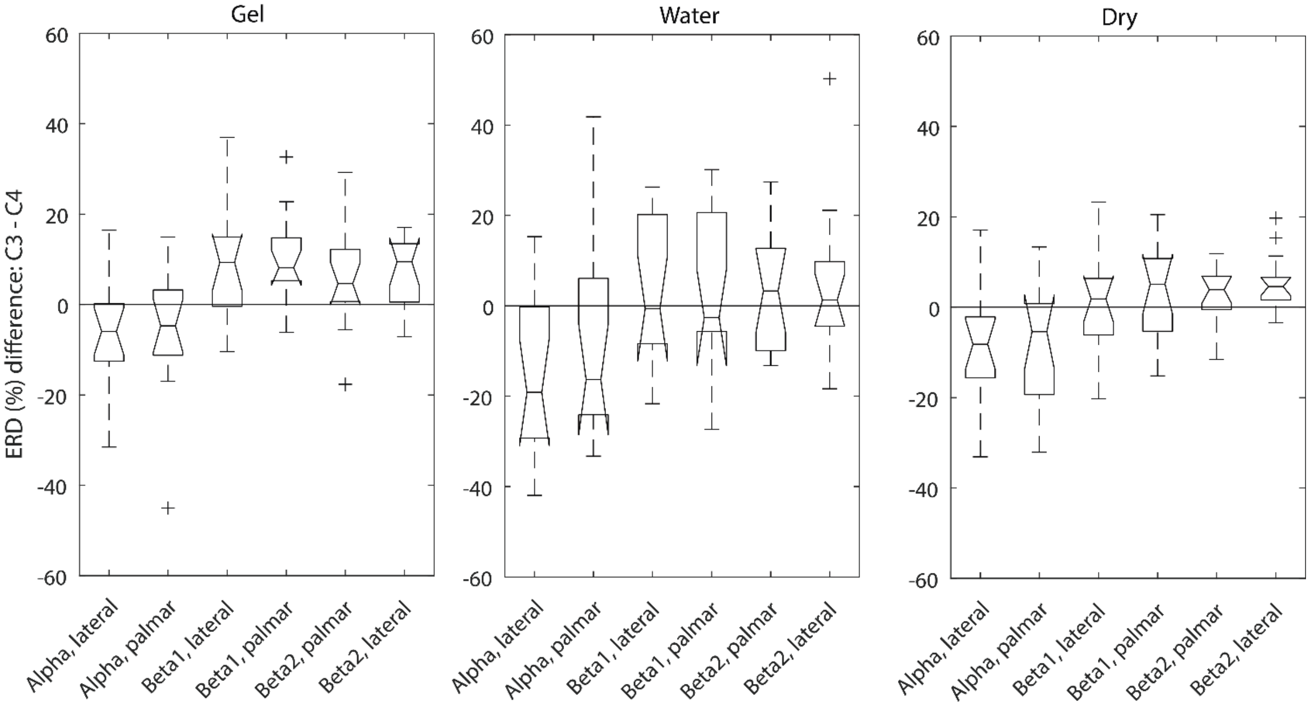

Figure S1: ERD lateralization in the three recordings. Box-plots of the ERD difference between C3 and C4. Positive values indicate a stronger contralateral desynchronization (also hotter colors in the time-frequency maps), whereas negative values indicate a stronger ipsilateral desynchronization.

In summary, a stronger ipsilateral ERD can be observed in alpha (mu) for the three recordings and movement conditions. Regarding beta lateralization, gel-based recordings show a contralateral lateralization, water-based recordings do not show a lateralization, and dry electrodes show a contralateral lateralization specifically in beta2.

2 Multiclass single-trial classification with reduced channels over the sensorimotor cortex

This section reports the gel- and water-based electrodes results of the multiclass single-trial classification when only using a subset of 11 channels covering the sensorimotor cortex, specifically those electrode positions of the dry-electrodes headset (EEG-Hero™): FC3, FCz, FC3, C3, C1, Cz, C2, C4, CP3, CPz, CP4.

Table S1: Participant-specific classification results of the gel-based recordings with reduced channels. Columns 2 to 4 show peak performance (%), standard deviation (%) and time of occurrence (s) with respect to the movement onset for the calibration set. Columns 5-7 show the same for the test set.

	**Calibration set**			**Test set**		
**#**	**Peak (%)**	**STD (%)**	**Time (s)**	**Peak (%)**	**STD (%)**	**Time (s)**
G01	66.3	7.5	1.6	59.5	8.3	1.6
G02	58.5	9.6	1.0	52.6	5.7	1.1
G03	58.1	8.1	2.1	51.2	9.0	1.9
G04	76.2	6.8	0.8	63.7	9.9	0.8
G05	62.0	9.1	1.3	54.5	7.4	-1.6
G06	57.6	8.9	0.9	46.3	5.8	-1.7
G07	45.9	9.1	0.7	49.4	5.6	0.6
G08	65.4	7.1	1.1	80.0	12.4	1.2
G09	72.3	7.0	0.8	58.5	7.3	0.8
G10	64.0	8.2	0.9	59.5	7.2	1.0
G11	53.7	9.1	0.6	52.5	6.4	0.6
G12	60.1	8.7	1.7	56.1	9.1	1.8
G13	51.9	8.0	0.3	58.5	8.2	0.3
G14	54.9	8.9	0.4	52.8	6.1	0.5
G15	63.3	8.1	0.8	66.3	8.9	0.9
**AVG**	**60.7**	**8.3**	**1.0**	**57.4**	**7.8**	**0.7**

Table S2: Participant-specific classification results of the water-based recordings with reduced channels. Columns 2 to 4 show peak performance (%), standard deviation (%) and time of occurrence (s) with respect to the movement onset for the calibration set. Columns 5-7 show the same for the test set.

	Calibration set			Test set		
**#**	**Peak (%)**	**STD (%)**	**Time (s)**	**Peak (%)**	**STD (%)**	**Time (s)**
V01	61.6	9.7	0.9	58.2	11.2	0.8
V02	67.1	7.3	0.6	67.9	8.2	0.8
V03	62.1	6.4	0.9	53.6	6.5	1.0
V04	58.0	8.0	0.3	50.6	6.9	0.4
V05	59.4	7.0	0.9	51.8	8.5	0.9
V06	73.3	7.8	0.9	76.3	11.8	1.0
V07	64.0	6.9	0.9	59.5	8.0	0.8
V08	72.9	7.7	1.2	52.0	6.0	0.7
V09	61.1	7.8	0.8	63.9	10.3	0.9
V10	61.2	9.6	0.8	58.0	8.1	0.9
V11	57.0	9.0	0.1	51.9	7.5	0.1
V12	55.7	7.6	-0.5	48.8	8.5	-0.3
V13	60.4	8.0	0.3	53.7	6.8	0.3
V14	58.5	8.6	1.0	56.4	9.7	0.6
V15	60.1	8.7	0.8	60.5	8.0	0.9
**AVG**	**62.2**	**8.0**	**0.7**	**57.5**	**8.4**	**0.7**
